# Lignin-Based High-Performance Fibers by Textile Spinning Techniques

**DOI:** 10.3390/ma14123378

**Published:** 2021-06-18

**Authors:** Yanhong Jin, Jiaxian Lin, Yu Cheng, Chunhong Lu

**Affiliations:** 1 Key Laboratory of Textile Science and Technology, Ministry of Education, Donghua University, Shanghai 201620, China; 2180045@dhu.edu.cn (Y.J.); 2190041@mail.dhu.edu.cn (J.L.); 2190053@mail.dhu.edu.cn (Y.C.); 2 College of Textiles, Donghua University, Shanghai 201620, China

**Keywords:** lignin, high-performance fibers, textile spinning techniques, mechanical performance

## Abstract

As a major component of lignocellulosic biomass, lignin is one of the largest natural resources of biopolymers and, thus, an abundant and renewable raw material for products, such as high-performance fibers for industrial applications. Direct conversion of lignin has long been investigated, but the fiber spinning process for lignin is difficult and the obtained fibers exhibit unsatisfactory mechanical performance mainly due to the amorphous chemical structure, low molecular weight of lignin, and broad molecular weight distribution. Therefore, different textile spinning techniques, modifications of lignin, and incorporation of lignin into polymers have been and are being developed to increase lignin’s spinnability and compatibility with existing materials to yield fibers with better mechanical performance. This review presents the latest advances in the textile fabrication techniques, modified lignin-based high-performance fibers, and their potential in the enhancement of the mechanical performance.

## 1. Introduction

Environmental concerns and emerging requests for sustainable and renewable resources have drawn intense attention to biopolymer-based products [1,2]. Biobased materials derived from the plants (i.e., starch, cellulose, lignin, alginate, etc.) and animals (i.e., chitin/chitosan, gelatin, wool, silk, collagen, etc.) are abundant and renewable, thereby having great potential to alleviate the current dependence on the expensive and nonrenewable petroleum-based products [3]. Moreover, biopolymers show desired properties (i.e., low density, high toughness, reasonable specific strength, good thermal properties, flexible processing procedure, biodegradability, recyclability, etc.), which make them suitable for extensive applications [4,5,6,7], such as reinforcement materials [8].

Next to cellulose, lignin is the second most abundant biopolymer in nature. Nearly 50 million tons of lignin are produced each year as a byproduct of pulp and paper industry, while only 2% is used for low- and medium-value applications. In fact, most lignin is currently utilized as low-value fuel for energy generation [9,10,11,12]. With outstanding properties (i.e., high carbon content (30–40 wt.%) [13,14], high thermal stability, biodegradability, favorable stiffness, etc.) and cost-effectiveness (low price ~3.08 USD/kg), lignin has the potential to be developed into value-added products for various applications [7,15,16]. For instance, lignin has been used in polymer composites as stabilizing agents, lubricants, coatings, plasticizers, surfactants, polymer-reinforced fillers, superabsorbent hydrogels, etc. [17,18,19,20,21,22,23]. The development of novel materials from lignin or lignin-derived products will promote the economics of polymer composite materials, as well as possibly address lignin’s disposal issue in the paper-making industry [24,25,26]. For polymer composite applications, an intensified focus has been directed toward the use of lignin-based carbon fibers and fiber reinforcement to potentially replace the petroleum-based plastics. In the 1960s, Otani et al. [27] prepared lignin-based carbon fibers with a diameter of 20–30 μm and a tensile strength (TS) of ~0.8 GPa, confirming the feasibility of lignin as a raw material for carbon fibers due to its aforementioned high carbon content. Moreover, the promise of lignin as a filler for fiber reinforcement and how to make the final product completely/partially biodegradable and ecofriendly have been stressed [7,12]. The effectiveness of biobased resources for high-performance applications relies heavily upon the composition, structure, and extraction methods of the raw materials [28]. Structure features (i.e., amorphous chemical structure, low molecular weight, and broad molecular weight distribution) and poor alignment of lignin are the critical factors limiting the enhancement of mechanical properties of lignin-based fibers. To address these issues, some investigations have been conducted on fractionation or pretreatments to regulate the quality and uniformity of the lignin [29,30], on chemical methods to alter lignin structure [31,32], or on integrating lignin with other polymers to enhance strength [33].

Moreover, the importance of utilizing the textile spinning technique to obtain lignin-based high-performance fibers should be emphasized. The aim of fiber spinning is to retain the strength of the biomass and to enhance the orientation of polymer chains along the axial direction for better mechanical properties of the ultimate biobased fibers. Spinning methods (i.e., melt spinning, wet spinning, dry spinning, etc.) have their peculiar characteristics. For example, melt spinning is related to raw materials with appropriate thermal properties, narrow molecular weight distribution, and enough mobility (acceptable viscosity range of 100–450 Pa·s [34]) for the spinning process [35]. Gel-spinning typically generates high-strength fibers with few voids, but the concentrated spinning dope and low coagulation temperature required may affect the spinnability [36]. For each spinning technique, spinning conditions (i.e., temperature, speed, lignin structure, molecular weight, etc.) have great influence on the spinnability and mechanical performance of final lignin-based fibers, which should be comprehensively summarized.

**Figure 1:**
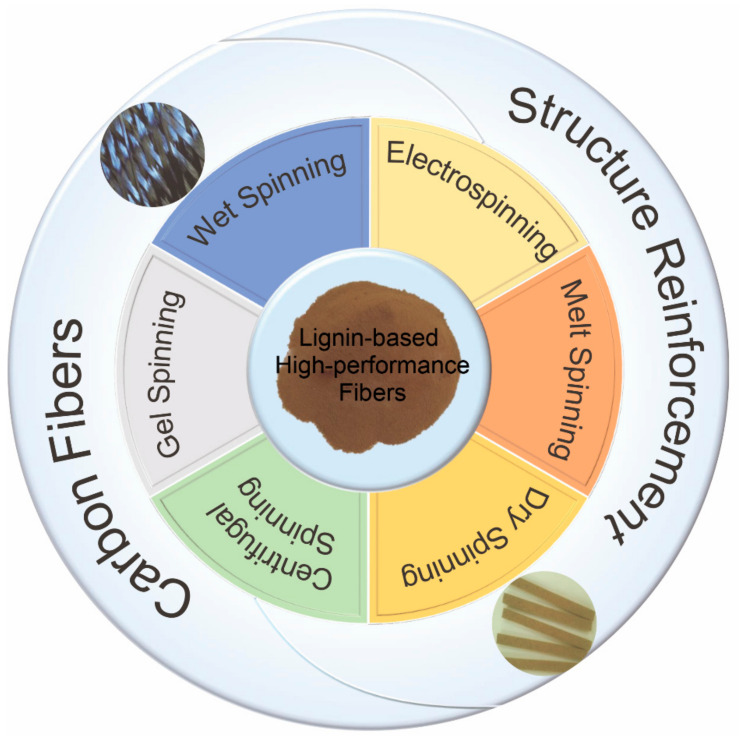
Diagram illustrating overview of lignin-based high-performance fibers for carbon fiber and structure reinforcement of composite (reproduced from an open-access article [45]) applications by textile spinning techniques in this review.

Several published review articles have summarized various carbon fiber precursors [37,38], different aspects regarding lignin pretreatment [39], fiber formation [40,41], applications [40], and the graphitic structure [42], surface treatment [43] of lignin-based carbon fibers or nanofibers [13,44], and lignin’s use in bulk composites [7,12]. However, incomplete information is available in the existing literature that comprehensively summarizes different textile fiber spinning techniques for fiber formation (Figure 1) of lignin-based carbon fibers and lignin-reinforced polymeric fibers potentially for composite applications and corresponding mechanical performance, which is vital for high-performance applications. Thus, this article reviews the feasibility of using lignin as an indispensable component to prepare low-cost biobased fibers including carbon fibers and lignin-reinforced polymeric fibers from various textile spinning techniques. In this review, an overview of different lignin types and corresponding structures and properties is first introduced. Moreover, recent advances regarding the spinnability and physical performance of lignin-based fibers including lignin-based carbon fibers and lignin-reinforced polymeric fibers from different spinning methods are discussed. Lastly, the review concludes with future perspectives on fabricating high-performance lignin-based fibers.

## 2. Lignin’s Structure and Properties

**Figure 2:**
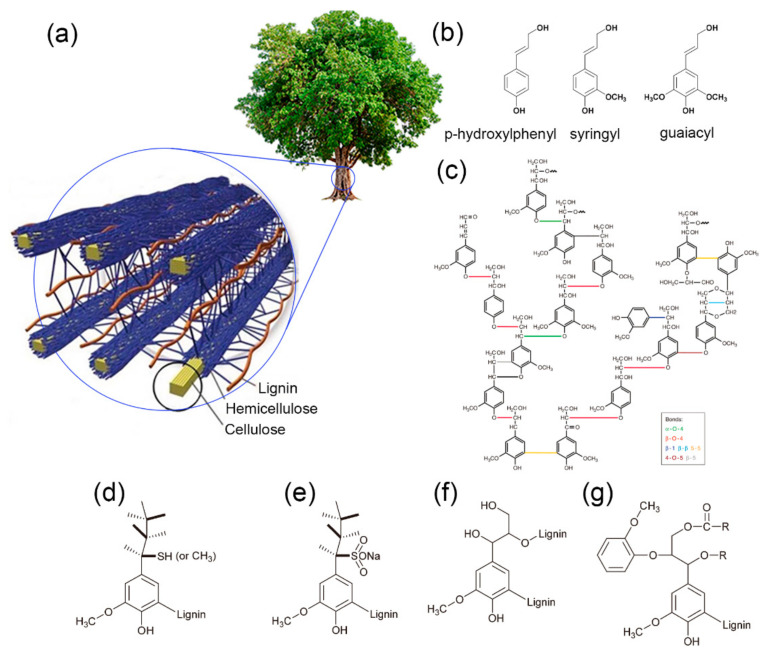
(**a**) The structure of lignocellulose biomass present in plants: highly aligned cellulose strands are surrounded by hemicellulose and lignin. (**b**) The chemical structure of three major monomers of lignin. (**c**) The general chemical structure of lignin with different C–O (β-*O*-4, 4-*O*-5, or α-*O*-4) and C–C (β-5, β–β, β-1, or 5-5) linkages. Technical lignins of (**d**) Kraft, (**e**) lignosulfonate, (**f**) soda, and (**g**) organosolv lignin. (**a**) Reproduced with permission from [46], copyright 2011 Elsevier. (**b**) Reproduced with permission from [55], copyright 2015 Royal Society of Chemistry. (**c**) Reproduced with permission from [56], copyright 2012 Elsevier. (**d**–**g**) Reproduced with permission from [52], copyright 2016 Royal Society of Chemistry.

Lignin is one of the main constituents in the cell walls of plants, together with cellulose and hemicellulose (Figure 2a) [46]. It is a biopolymer with highly branched polyphenolic network structures [46]. The monomers of lignin are phenyl propane units that differ in the degree of oxygen substitution on the phenyl ring (Figure 2b). The H-structure (*p*-hydroxy phenyl) has a single hydroxyl or methoxy group, the G-structure (guaiacyl) has two such groups, and the S-structure (syringyl) has three. Those three monomers are linked by various types of C–O (β-*O*-4, 4-*O*-5, or α-*O*-4) and C–C (β-5, β–β, β-1, or 5-5) bonds (Figure 2c). The predominant linkage in the structure is β-*O*-4, which constitutes more than 50% of the linkage units in both softwoods and hardwoods [47]. Hydrogen bonding between adjacent carboxylic, hydroxyl, and ether groups, as well as π–π interactions between aromatic moieties [48,49,50], accounts for the three-dimensionally (3D) branched and complex structures of lignin. Due to lignin’s complex interconnected structure, the exact chemical structure and molecular weight of lignin are still unknown, and there is enormous variation in the structure depending on the sources and isolation methods [51]. Although lignin contains a large number of hydroxyl groups, it is considered more hydrophobic than carbohydrate polymers (cellulose and hemicellulose) in plants. Thus, lignin plays an important role in plants, providing mechanical support, regulating water transport, and protecting the living plants against microorganisms [52,53]. On the basis of the abovementioned structure features, lignin is regarded as a potential source of phenolic compounds to replace petroleum-based chemicals and to manufacture high-value-added chemical products [54].

In general, wood-derived lignin can be categorized into hardwood or softwood lignin according to its source. Lignin in softwood contains almost 90% G-units [54], whereas hardwood lignin is mainly composed of G- and S-units, with higher S-unit proportions; it demonstrates thermoplastic characteristics with a definable melt-processable temperature [57]. Moreover, softwood and hardwood lignins demonstrate a huge difference in how the monomers are connected. Compared with hardwood lignin, softwood lignin has more resistant C–C (β–β, β-5, β-1, and 5-5) bonds and a lower content of ether bonds because the C_5_ positions of G-units in softwood lignin tend to involve a coupling reaction to form resistant linkages, whereas those in S-units are sterically inhibited due to the additional methoxy group (OCH_3_) [58,59]. In other words, softwood lignin readily forms dibenzodioxocin [60], while hardwood lignin tends to form a more linear structure than softwood lignin due to the excessive number of S-units in the structure [57]. The difference in chemical structures of softwood and hardwood lignins leads to remarkably different softening temperatures, which are 138–160 °C for softwood lignin and 110–130 °C for hardwood lignin [61]. The softening temperature of lignin is defined as the temperature at which the internal friction of lignin is at a maximum value [62]. At the softening temperature or glass transition temperature (T_g_), polymers achieve sufficient energy to decrease the mutually attractive forces, such that they become rubbery or plastic to a greater or lesser degree [63]. It is this softening behavior of lignin at elevated temperature that has inspired research efforts to develop lignin-based high-performance fibers to replace currently ubiquitous petroleum-based high-performance fibers.

Lignin can also be classified into four different types of technical lignin, according to the pulping process: Kraft, lignosulfonate, soda, and organosolv lignin. Firstly, most lignin is extracted by the Kraft process, which accounts for the largest volume among all types of lignin. In this process, lignin’s linkages or covalent attachments to surrounding materials of cellulose and hemicellulose are degraded in the presence of white liquor, which is an aqueous solution of sodium hydroxide (NaOH) and sodium sulfide (Na_2_S) mixture. Thus, the Kraft process fractures lignin into smaller and water-soluble molecules, allowing further fragmentation of lignin as the linkages between the phenylpropane units are cleaved [47]. Structurally, Kraft lignin has aliphatic thiol groups (Figure 2d), which make the biopolymer hydrophobic, and it contains sulfur (1–2 wt.%) [46] after the Kraft pulping process. Second, the sulfite process is quite similar to the Kraft process, except that the medium of the former is acidic. In the sulfite pulping process, salts (such as sodium (Na^+^), potassium (K^+^), ammonium (NH^4+^), calcium (Ca^2+^), and magnesium (Mg^2+^) counterions) of sulfurous acid aqueous solution, sulfites (SO_3_^2−^), or bisulfites (HSO_3_^2−^) degrade and sulfonate the lignin by replacing the hydroxyl group with a sulfonate group. As a result, lignin is solubilized and separated from the cellulose in non-precipitated form. This process can be performed in the pH range of 2–12, depending on the cationic counterions. In most cases, this process is completed in acidic conditions with calcium or magnesium counterions [64]. The final product of lignin is in the form of lignosulfonates [65,66]. The use of sulfurous acid in the pulping process yields sulfonate groups (–SO_3_) in the chemical structure of lignosulfonates (Figure 2e), making them water-soluble over a wide range of pH values [46]. Thirdly, soda lignin undergoes a similar cooking process to Kraft lignin, except that soda pulping does not contain Na_2_S in the alkali medium [67]. Due to the absence of a strong nucleophile, depolymerization of lignin with only alkaline (NaOH) is less efficient [67] in comparison with the Kraft process. This soda pulping process is a predominant method for chemical pulping of non-wood species such as bagasse, wheat straw, hemp, kenaf, and sisal [68]. With no sulfur (Figure 2f) and little hemicellulose contaminating its structure, soda lignin can be used directly without purification. These features make soda lignin more suitable for chemical modification in order to be used in other value-added applications. Lastly, organosolv pulping is a process that uses organic solvents (i.e., acetic acid, formic acid, ethanol, peroxiorganic acids, etc.) and water as the cooking liquid for the wood chips [69]. Organosolv lignin, like soda lignin, has little modification of structure after being recovered from the organosolv pulping process. Organosolv lignin has a lower softening temperature (90–110 °C) than soda lignin (140 °C) [52]. The former contains fewer aliphatic hydroxyl groups (Figure 2g) than soda lignin. This is due to the organosolv process involving the depolymerization of lignin by acid-catalyzed cleavage of β-ether linkages [70]. The produced lignin is abundant in β-ether linkages, while the fragments can condense back to higher-molecular-weight polymers at lower pH via the elimination of CH_2_OH [71]. Organosolv lignin is in a relatively pure state, which is ideal for direct usage to manufacture different products.

## 3. Mechanical Performance of Lignin-Based Fibers

Textile fiber spinning is a typical process that involves the orientation of polymer melts or solutions to align the macromolecules and as a result increases the strength of the material [72]. In the textile industry, fiber spinning techniques are widely used in the production of synthetic and regenerated fibers, and they generally include conventional spinning techniques (such as melt spinning, wet spinning, dry spinning, and gel spinning), as well as electrospinning and centrifugal spinning. Conventional fiber spinning techniques often yield polymeric fibers with diameters in the micrometer range [73]. Electrospinning and centrifugal spinning are capable of consistently producing fibers with diameters down to several nanometers [74,75].

In general, lignin is usually structurally modified, blended with other thermoplastic polymers, or added as a reinforcement material to enhance the fiber performance. In this section, the efforts made to improve the spinnability and mechanical performance of lignin-based high-performance fibers obtained via various spinning techniques are discussed.

### 3.1. Lignin-Based Carbon Fibers

As one type of high-performance fiber, carbon fibers are used as reinforcement in various composites [76]. Among the common precursors for commercially available carbon fibers, petroleum-based polyacrylonitrile (PAN) is the dominant precursor, which is quite expensive and accounts for more than 50% of the overall cost of carbon fiber production. Other precursors, such as pitch and rayon, are either too expensive or yield carbon fibers with less satisfactory mechanical properties [77]. Lignin has drawn intense attention as a potential carbon fiber precursor due to its natural abundance and high carbon yield with its unique aromatic structure. Lignin-based carbon fibers have extremely low cost and long-term sustainability if precursor-grade lignin is successfully converted into fibers.

#### 3.1.1. Melt-Spun Lignin-Based Carbon Fibers

**Figure 3:**
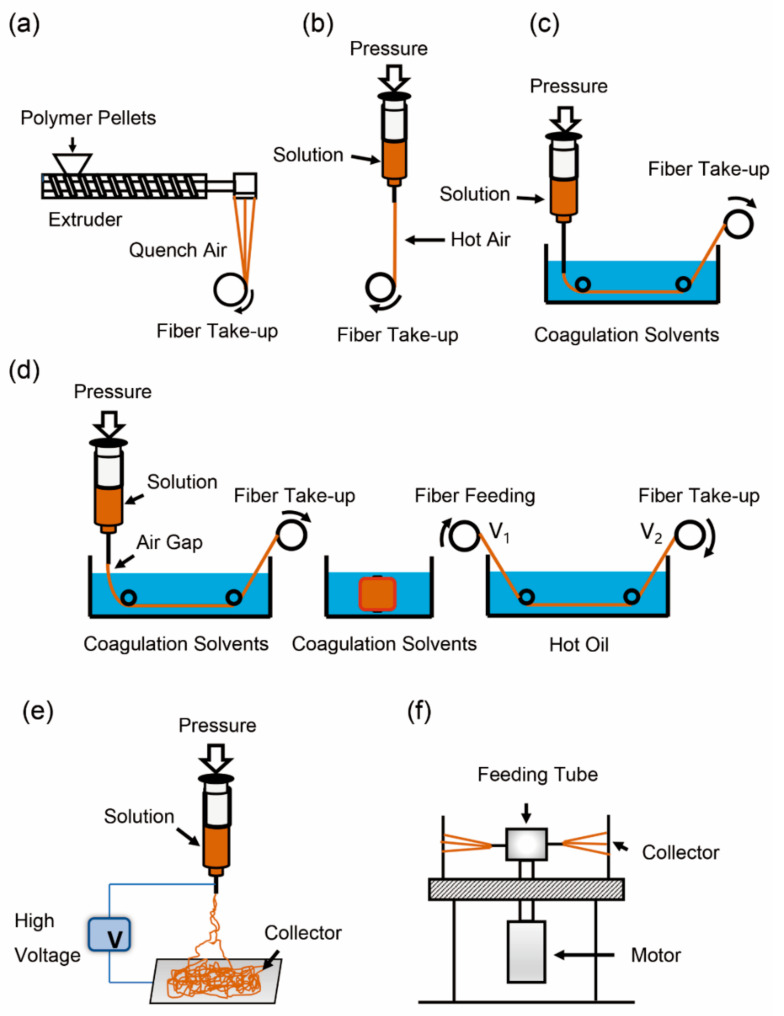
Simplified processes for (**a**) melt spinning, (**b**) dry spinning, (**c**) wet spinning, (**d**) gel spinning, (**e**) electrospinning, and (**f**) centrifugal spinning.

Melt spinning of lignin-based fibers has drawn intense attention since the 1990s [33,78,79,80,81,82,83,84,85]. This high-speed process can potentially produce low-cost lignin-based carbon fiber precursors. Generally, melt-spun lignin-based fibers are achieved by converting lignin powders or pellets into fibers or filaments above the softening temperature, and then quenching them in cold air before fibers are collected at high speed (Figure 3a). The mechanical properties of melt-spun lignin fibers mainly depend on different factors such as the source of lignin (molecular weight, softening temperature, molecular structure), degree of crystallinity, and orientation of the fiber, which are closely related to processing parameters of the melt spinning process (i.e., heating temperature, extrusion speed, take-up speed, etc.) [86,87].

It should be noted that most lignins have small molecular weight and no melting point due to the amorphous chemical structure; thus, they often decompose when heated at a temperature higher than 200–250 °C [88], which greatly affects the processing temperature. The difference between the extrusion speed and collecting speed is vital in increasing the degree of molecular orientation and percentage crystallinity, which contribute to the mechanical performance of the fibers [89]. However, lignin’s amorphous, three-dimensionally branched structure prevents the formation of crystals in the fiber spinning process, which may hinder fiber strength. The difference in lignin chemical structure due to source also affects melt processability. For instance, the higher softening temperature of softwood lignin often leads to poorer spinnability by melt spinning, thereby requiring additional treatments such as chemical modification or blending with other polymers for fiber processing. Even though hardwood lignin is generally melt-processable, the ratio between S- and G-units should be carefully tuned for efficient melt spinning. Moreover, it is difficult to stabilize melt-spun lignin precursor fibers to yield low-cost carbon fibers via thermo-oxidation since they are prone to partially fuse during the stabilization and carbonization process [84]. This is mainly due to the melt-spun lignin-based precursor fibers lacking sufficient chemical reactivity to be crosslinked during the stabilization process before undergoing the ultimate high-temperature carbonization process. As a result, melt-spun lignin-based carbon fibers have structural defects and inferior mechanical performance to PAN-based carbon fibers [31].

To improve fiber spinnability, it is of great importance to modify lignin to achieve some extent of molecular rotation and adequate material fluidity for melt-spun lignin-based fibers [78]. Lignin modifications could reduce the amount of hydroxyl groups or crosslinks that resist flow to make lignin a more flexible polymer at high temperatures of the melt spinning process [78]. Another commonly used method to improve spinnability is to blend lignin with other polymers (i.e., polyethylene (PE), polypropylene (PP), poly(ethylene oxide) (PEO), poly(ethylene terephthalate) (PET), etc.), and later melt-spin the blend into fibers with no porous structure but highly rough surfaces [90]. Unfortunately, poor compatibility of lignin and polymer in melt spinning becomes the main obstacle that hinders the enhancement of the composite fiber’s mechanical properties. Table 1 summarizes the characteristics of melt-spun lignin-based carbon fibers from the reported literature, which are presented in detail below.

##### Structurally Modified Melt-Spun Lignin-Based Carbon Fibers

Kadla et al. [82] first reported the production of lignin-based carbon fibers from a commercially available hardwood Kraft lignin by melt spinning with no modification. The obtained carbon fibers had relatively low mechanical performance of 422 MPa for tensile strength (TS) and of 40 GPa for tensile modulus (TM). Generally, it is easier to melt-spin hardwood lignin than softwood lignin due to the difference in chemical structures as mentioned above. In detail, softwood lignin contains more hydroxyl groups than hardwood lignin, which allows the former to easily crosslink via intramolecular interactions and lose fluidity at high temperature of the melt spinning process. To essentially enhance the spinnability and mechanical performance of lignin-based fibers with altered lignin structure, several common strategies may be used.

One strategy is to increase lignin’s molten fraction by modifying its molecular weight. By investigating the influence of molecular weight and fusible fractions on the spinnability of softwood lignin-based melt-spun fibers, it was shown that the weight-averaged molecular weight (M_w_) should be less than 3,200 Da or the fusible fraction should be ~58.2% to achieve molten lignin for melt spinning [100]. In most cases of the melt-spun lignin fibers, lignin purification and fractionation, by ultrafiltration [96], pH-fractionation [104], and solvent extraction [70,97,105], are used to obtain lignin with high thermal mobility and improved performance. Essentially, purification and fractionation [106,107,108] can isolate a lower-molecular-weight part (and correspondingly a lower T_g_) of lignin for better processability by melt spinning. For instance, a corn-stover lignin fractionated with methanol had significantly lower M_w_ (1,200 Da) and polydispersity index (PDI = 1.89) than raw lignin (3,263 Da and 2.91, respectively) [97]. Although low-molecular-weight lignin facilitates the melt spinning process at low temperature, it causes fiber fusion problems in the stabilization process due to its low T_g_. Thus, the fractionated lignin or low-molecular-weight lignin were thermally treated to allow repolymerization [98] to increase thermal stability and then acetylated to yield melt-spun carbon fiber with TS = 0.45 GPa and TM = 62 GPa, respectively [97]. Moreover, thermal treatment of lignin before spinning was used to achieve pyrolytic lignin with low molecular weight to facilitate melt spinning and to maintain fiber integrity during the stabilization and carbonization processes [91]. The resulting carbon fibers had relatively low TS = 370 MPa and TM = 36 GPa due to large fiber diameter (49 μm) and voids. Furthermore, pyrolytic lignin recovered from hardwood by a phenolic bio-oil was catalytically repolymerized to yield suitable molecular weight for melt-spinnable precursor fibers and resulting carbon fibers with TS = 850 MPa and TM = 85 GPa [98]. Ideally, lignin-based carbon fiber precursors should have a high molecular weight and low PDI to achieve structural uniformity [41] and good mechanical performance. However, this contradicts with the need for the good processability of melt spinning with low-molecular-weight lignin. Thus, selection of low-molecular-weight lignin often facilitates fiber processing but yields lignin-based carbon fibers with only moderate mechanical performance.

**Figure 4:**
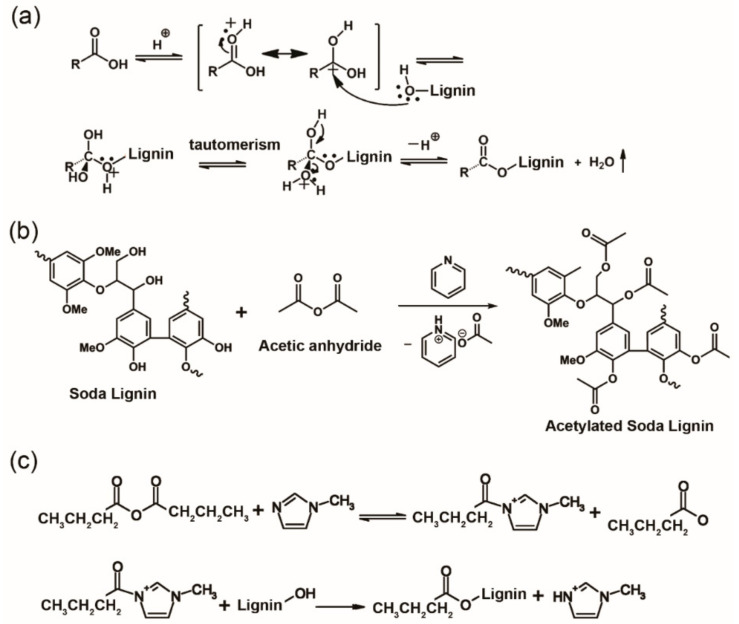
The chemical reaction mechanisms of (**a**) direct esterification, (**b**) acetylation, and (**c**) butyration of lignin. (**a**) Reproduced with permission from [109], copyright 2019 Royal Society of Chemistry. (**b**) Reproduced with permission from [110], copyright 2016 American Chemical Society. (**c**) Reproduced with permission from [93], copyright 2005 American Chemical Society.

Another important strategy is to chemically modify lignin’s structure to allow fluidity at the high-temperature of melt spinning. The most commonly used chemical modification methods including esterification [109], acetylation [110], and butyration [93] (Figure 4), while other methods (i.e., hydrogenolysis, phenolysis, solvolysis) are also used to yield modified lignin. Sudo et al. [78] reported hydrogenated steam-exploded lignin. Structurally, the alkaline hydrolysis of lignin involved the cleavage of alkyl–aryl ether bonds and formation of ethylene bridges (–C=C–) between aromatic rings, as well as the reduction of hydroxyl groups (–OH) and removal of methylol groups (–CH_2_OH). With a vacuum treatment at 300–350 °C, the melt viscosity of hydrogenated lignin was improved to facilitate the melt spinning process. The carbon fibers prepared from this modified lignin exhibited a diameter of 7 μm, a TS of ~660 MPa, and Young’s modulus of ~40.7 GPa, but a low yield (<20%). Sudo et al. [85] further obtained melt-spun phenolated steam-exploded lignin as carbon fiber precursors. This alternative modification method was developed because of its lower cost and higher yield (>40%) than hydrogenated lignin. Lignin and phenols with alkyl groups were reacted at 180–300 °C for 2–5 h in the presence of a catalyst. Crosslinks between lignin aromatic groups were reduced, and the thermal viscosity of lignin was increased to enhance the melt spinnability of lignin with a lower softening temperature range of 150–190 °C. However, the relationship between the thermal properties of modified lignin and the degree of phenol substitution was not revealed. The resulting carbon fiber displayed a TS of ~455 MPa, which was worse than that of hydrogenated lignin. Softwood lignin obtained by solvolysis of wood chips using polyethylene glycol (PEG) and sulfuric acid had good flow properties for melt spinning due to the PEG moiety linked to the lignin structure [103]. However, the stabilization of the precursor fibers was difficult, and the resulting carbon fibers (fiber diameter ~10 μm) had a low TS (~457 MPa) and TM (~27 GPa) due to the porous and flabby structure of the fibers.

Partially acetylated lignin was isolated by aqueous acetic acid pulping [92]. Lignin-based carbon fibers were melt-spun from organosolv lignin, which was obtained from aqueous acetic acid pulping without further modification. The isolated lignin could be successfully spun into fibers due to the narrow PDI and partial acetylation of the hydroxyl groups in lignin during the pulping process. The key was that acetylation (Figure 4b) would remove hydroxyl groups and crosslinks that resist flow during the melt spinning process, rendering lignin a more flexible polymer. The spinnability improved with an increasing degree of acetylation. However, the mechanical performance of the converted carbon fibers was not very satisfactory, which was 355 MPa for TS and 39.1 GPa for TM with a fiber diameter of 14 μm. The low mechanical performance was due to the rough and porous fiber structure since the melt spinning of partially acetylated lignin at high temperature (>300 °C) could have resulted in partial decomposition or degradation of the products. Nevertheless, this partially acetylated lignin from acetic acid pulping had the potential for generating activated carbon fibers by melt spinning [92].

Most recently, lignin-based acrylate polymers were achieved via a two-step functionalization of lignin bio-oil followed by radical polymerization [99]. The orientable new polymer was melt-spun and converted into carbon fibers with an average TS of ~1.70 GPa and TM of ~182 GPa. This is the melt-spun lignin-based carbon fiber with the best physical properties reported up to date due to its high degree of graphitization and low number of structural defects.

It is remarkably challenging to produce melt-spun lignin-based carbon fibers with satisfactory mechanical performance for composites applications (i.e., at least TS = 2 GPa and TM = 200 GPa are required for PAN-based carbon fibers) [111]. The key technical difficulty lies in that it is hard for lignin fiber precursors to possess good chemical stability for spinnability in fiber spinning but low or adequate chemical reactivity to internally crosslink and yet not stick to each other during the stabilization process [36]. In addition, it is contradictory to allow lignin have T_g_ that is low enough to ease the melt spinning process but high enough to shorten the stabilization time [88].

##### Melt-Spun Lignin/Polymer Blend Carbon Fibers

Apart from structural modification of lignin, blending lignin with polymers (i.e., PP, poly(vinyl alcohol) (PVA), PAN, polylactic acid (PLA), PEO, etc.) is an effective and low-cost method to improve the spinnability of lignin-based carbon fibers. Several factors that may influence the physical properties of fibers from lignin/polymer blends should be noted: source and properties of lignin, weight fraction and inherent properties of polymer, and the interactions between lignin and polymer.

**Figure 5:**
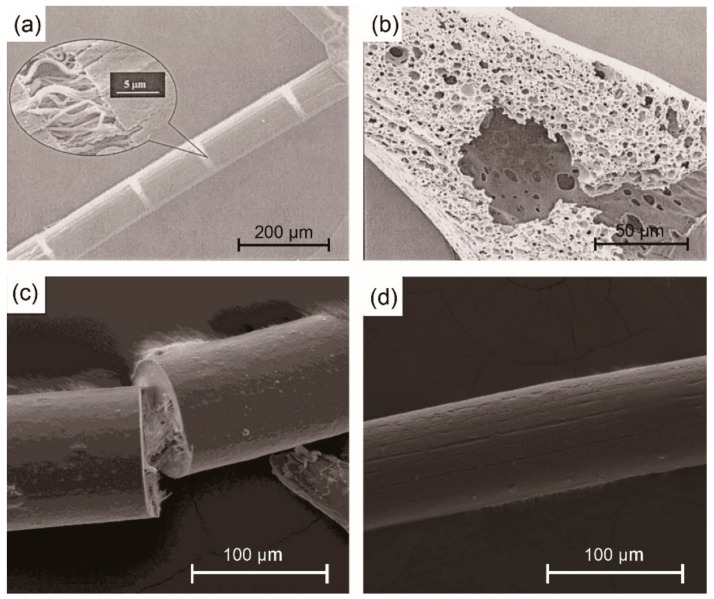
Scanning electron microscopic (SEM) images of melt-spun lignin/PP (*w/w* 75/25) fibers: (**a**) as-spun fibers after stretching to produce fractures along the fiber axis and (**b**) thermally treated fibers after being cut at a 30° angle with respect to the fiber axis; (**c**) butyrated lignin/PLA fiber (*w/w* 75/25) and (**d**) corresponding lignin/PLA carbon fiber by melt spinning. (**a**,**b**) Reproduced with permission from [113], copyright 2002 John Wiley and Sons. (**c**,**d**) From [101], an open-access thesis.

An earlier example of a lignin/polymer mixture for fiber melt spinning is the lignin/PEO system [82] with strong hydrogen bonding. The addition of PEO improved the moldability and flexibility of lignin, as well as fiber spinnability. The best mechanical properties were obtained by lignin/PEO (97/3, *w/w*) carbon fibers with TS = 0.55 GPa and TM = 60 GPa. However, with greater than 5 wt.% PEO, the lignin/PEO fiber was thermally unstable and fused together during carbonization. Kubo et al. [33] produced melt-spun carbon fibers from hardwood lignin blended with two recyclable polymers, PET and PP. The miscibility of lignin/PET blend was good due to the strong hydrogen bonding [112]; thus, the lignin/PET carbon fibers had a smooth surface and good mechanical properties (TS = 0.7 GPa, TM = 94 GPa). However, the lignin/PP blends were not miscible, which often resulted in phase separation (Figure 5a), porous carbon fiber structure (Figure 5b), and inferior mechanical properties (TS = 0.4 GPa, TM = 54 GPa).

**Figure 6:**
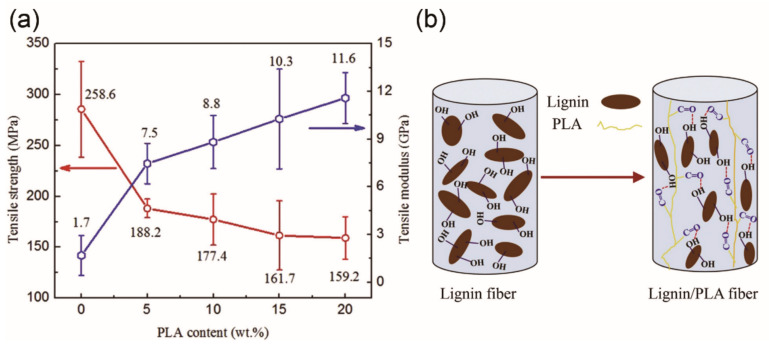
(**a**) Tensile strength and tensile modulus as a function of PLA contents for lignin/PLA-based carbon fibers and (**b**) schematic diagram of the orientation of lignin and lignin/PLA fibers. (**a**,**b**) Reproduced with permission from [80], copyright 2015 Elsevier.

Another polymer of interest for melt spinning with lignin is PLA. Chemically modified lignin from butyration (Figure 4c) was mixed with PLA to fabricate bio-renewable and low-cost carbon fibers [83]. The precursor fibers showed enhanced melt spinnability but poor mechanical properties. Lignin loading was varied at 0%, 50%, 75%, and 90% to PLA polymer. The TS of precursor fibers significantly decreased from 34 MPa of neat PLA to less than 5 MPa of 90% lignin/PLA fiber, and the Young’s modulus decreased from 26 GPa to 16 GPa. The inferiority of melt-spun composite fibers was mainly caused by poor compatibility or phase separation between modified lignin and PLA polymer (Figure 5c) [101]. Unfortunately, the mechanical performance of converted carbon fibers was not reported. Later, Wang et al. [80] reported lignin/PLA-based carbon fibers having an optimal TS = 0.25 GPa and TM = 11.6 GPa (lignin/PLA mass ratio of 80/20) by melt spinning (Figure 6a). It was believed that the interaction between lignin and PLA was responsible for the increased modulus of the blend-based carbon fibers relative to that from pure lignin (1.7 GPa). The orientation of lignin chains along the fiber axis was low and the interaction between lignin clusters was fairly weak, corresponding to a low TM (1.7 GPa) of these lignin-based carbon fibers. When PLA was blended with lignin during the melting process, hydrogen bonds were formed between the chains of PLA and lignin. In the blends, PLA chains were oriented along the fiber axis due to its linear structure in the fiber spinning process. The oriented PLA chains promoted lignin alignment along the fiber axis with the aid of formed hydrogen bonding between the two materials (Figure 6b), leading to an improved orientation of lignin phase and an increased TM of carbon fibers. However, the volatilization of PLA during thermal stabilization and carbonization caused voids on the surface and cross-section of the fibers, thereby deteriorating the TS of the lignin/PLA carbon fibers. Other blending polymers such as PET [102] and thermoplastic polyurethane (TPU) [94] have been also blended with fractionated or chemically modified lignin to fabricate lignin-based carbon fibers, whose properties are shown in Table 1.

Furthermore, different sources or types of lignin have been blended, for example, by plasticizing an infusible lignin with a fusible lignin, to enhance melt spinnability and prevent the miscibility problem in lignin/synthetic polymer blends. For instance, carbon fibers could be melt-spun from softwood Kraft lignin, which was originally infusible and later plasticized with fractionated hardwood Kraft lignin [96]. It is reported that organosolv hardwood lignin, with a lower number of aliphatic hydroxyl groups and phenolic acids, has better spinnability and yields stronger fibers with fewer defects [95]. Switchgrass lignins are found to be difficult to be converted into fibers with no defects for high tensile properties. However, its higher number of G-units and more condensed structure often lead to faster stabilization [70]. Accordingly, different ratios of organosolv switchgrass and yellow poplar (organosolv hardwood) lignin blends were capable of producing melt-spun lignin-based carbon fibers with no phase separation at higher stabilization rate (0.5 °C/min) [95]. However, the mechanical performance of the resulting carbon fibers was not satisfactory, with optimal values of TS = 0.52 GPa and TM = 38 GPa.

**Figure 7:**
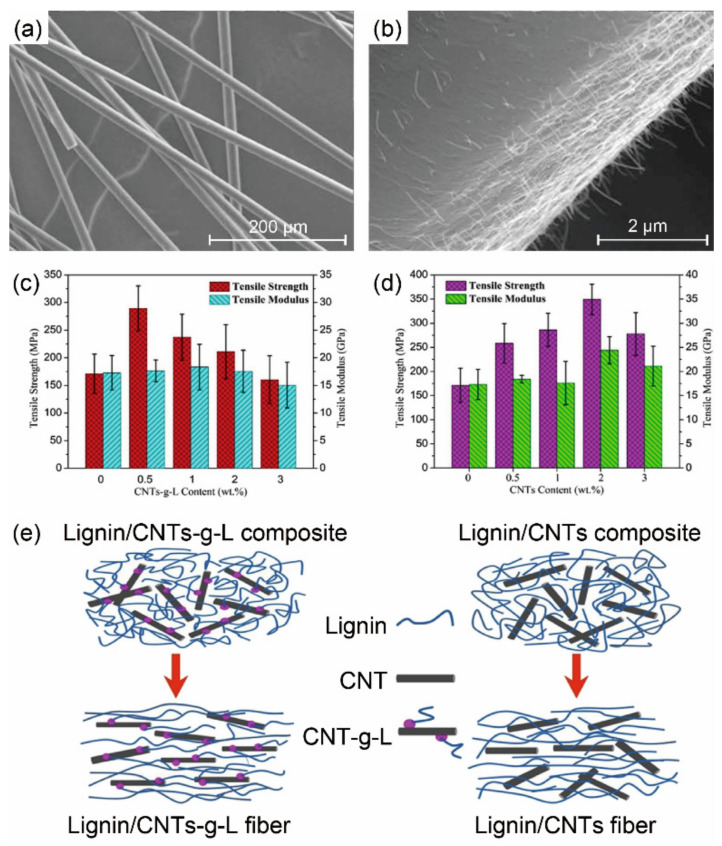
SEM images of 2.5% CNT/lignin fibers (**a**) before and (**b**) after fracture. (**c**) Mechanical properties of lignin/CNTs-g-L- and (**d**) lignin/CNT-based carbon fibers and (**e**) schematic diagram of the orientation of CNTs-g-L and CNTs. (**a**,**b**) Reproduced with permission from [41], copyright 2013 John Wiley and Sons. (**c**–**e**) Reproduced with permission from [79], copyright 2016 Elsevier.

Carbon nanotubes (CNTs) have been incorporated into lignin to achieve melt-spun carbon fibers [114]. At low CNT concentration (<15%), CNTs facilitated fiber spinnability by increasing heat capacity of the composite fibers, which made the fiber remain molten for longer distance along the spin-line and increased fiber stretching for finer fibers with smooth surface (Figure 7a). However, the interfacial adhesion between the non-functionalized CNTs and the lignin-based carbons was too weak (Figure 7b) to cause significant increase in mechanical performance of carbon fibers. A compatibilizer by grafting lignin chains onto the surface of CNTs (CNTs-g-L) was used to prepare lignin/CNTs-g-L fibers via melt spinning [79]. With 0.5% CNTs-g-L incorporated, the TS of lignin-based carbon fibers increased from 171.2 MPa to 289.3 MPa. The strong interactions between the CNTs-g-L and lignin increased the thermal stability of lignin but disordered the graphite structure of the carbon fibers. Due to the weaker interaction between CNTs and lignin, the contribution of the CNTs to the orientation phenomenon was less effective than that of CNTs-g-L, leading to an inferior TS (258.2 MPa) to that of lignin/CNTs-g-L-based carbon fibers with 0.5% fillers (Figure 7c,d). Therefore, the lignin/lignin or grafted lignin/lignin blends had enhanced melt spinnability but still low mechanical properties, probably due to the less linear structure of lignin and lack of stretching in the melt spinning process.

In summary, the compatibility of blending polymer with lignin is of great importance for mechanically enhancing lignin/polymer-based carbon fibers. Due to the phase separation between lignin and polymer, the porous structure resulting from the volatile fragments of polymers [33,83,115], and the insufficient stretching of the fibers for chain orientation, the melt spinning technique is not the best option to produce homogenous lignin/polymer carbon fibers with good mechanical performance.

#### 3.1.2. Solution-Spun Lignin-Based Carbon Fibers

Solution spinning is used to spin fibers from solutions of the dissolved polymer. It can be divided into three spinning techniques, dry spinning, wet spinning, and gel spinning, which all involve the preparation of homogenous spinning dopes. Solution-spun fibers generally have better mechanical performance than melt-spun fibers mainly due to the higher draw ratio of fibers.

As one type of the solution spinning technique, dry spinning yields fibers from polymer solutions after the evaporation of the volatile solvents [31,116]. The polymer solutions are first extruded through spinneret orifices and then into hot air for instant evaporation of the solvent. The solidified fibers or filaments may undergo further processing such as drawing before being collected onto a winder (Figure 3b). Unfortunately, the literature about dry-spun lignin-based fibers is quite limited [27,31,32,88,116]. This is possibly due to the higher cost and lower production efficiency of dry-spun fibers in comparison with melt-spun fibers. Moreover, dry spinning of lignin fibers may lead to voids and residual solvents within the fiber structure, which impede the fiber’s mechanical performance. One benefit is that dry spinning of lignin-based carbon fibers may circumvent the fusion issue of melt-spun lignin fibers during thermal treatment for carbonization and yield fibers with enhanced performance.

The preparation of lignin-based fibers via wet spinning has been shown to be a promising way to obtain low-cost fibers [117,118,119]. In conventional wet spinning, the polymer solution is directly extruded into a coagulation bath. The polymeric jets are in contact with the coagulants, which are miscible with the solvent but do not dissolve the polymer. The coagulated fibers are then collected onto a take-up roller (Figure 3c), which has variable speeds to stretch the fibers [119]. A common variation of wet spinning is dry-jet or air-gap spinning, which is often used for highly viscous polymer spinning dopes [120]. In this process, the spinning dopes are first extruded into an air gap of 10–200 mm [121], where the polymer jets are stretched to promote better molecular orientation before entering into a liquid coagulation bath. Other processing stages are similar to those of conventional wet spinning. Due to the relatively low molecular weight of lignin, it is hard to form solution viscous enough for wet spinning. Thus, high-molecular-weight polymers, such as PVA or PAN, are often mixed with lignin to improve the spinnability of wet-spun lignin/polymer solutions. Different factors such as solution concentration (including lignin/polymer weight ratio, molecular weight, and PDI of each component, solvent type, and temperature), coagulation bath (composition and temperature), and fiber draw ratio influence the morphology, microstructure, and mechanical properties of lignin-based fibers from wet spinning.

Gel spinning is a recently developed fiber spinning technique for producing lignin-based high-performance fibers. This process is similar to wet spinning. The hot polymer solution is extruded through a spinneret into an air gap, before being placed into a cooling coagulation bath to form as-spun gel filaments. The resulting as-spun gel fibers are collected onto a rotating winder and later immersed in the coagulation bath for a certain duration of time. The fibers then pass through the air oven or hot oil, where they are heated and stretched to form high-performance solid fibers (Figure 3d). Typically, gel-spun fibers have higher draw ratios than fibers processed by other fiber spinning techniques. This is because the lower chain entanglements and remaining solvents in the gel structure promote chain stretching in the drawing process [122]. Lignin is mainly blended with PAN or PVA, which are common polymers used in gel-spun fibers [123,124,125,126]. Several factors that influence the mechanical performance of gel-spun lignin-based fibers should be taken into consideration. For instance, polymer molecular weight [122], polymer concentration [127], and solvent removal [127,128] may affect fiber spinnability and fiber microstructure. Other factors such as the coagulation temperature, spinning dopes, coagulation solvent compositions, and fiber gelation time also impact the strength of gel-spun fibers [129]. Table 2 concisely summarizes the experimental conditions (materials, spinning parameters, thermal treatment) and key properties (fiber diameter and mechanical properties) of solution-spun lignin-based carbon fibers from the reported literature. Below, a more detailed account of the fabrication methodologies of these lignin-based carbon fibers is presented.

##### Structurally Modified Solution-Spun Lignin-Based Carbon Fibers

**Figure 8:**
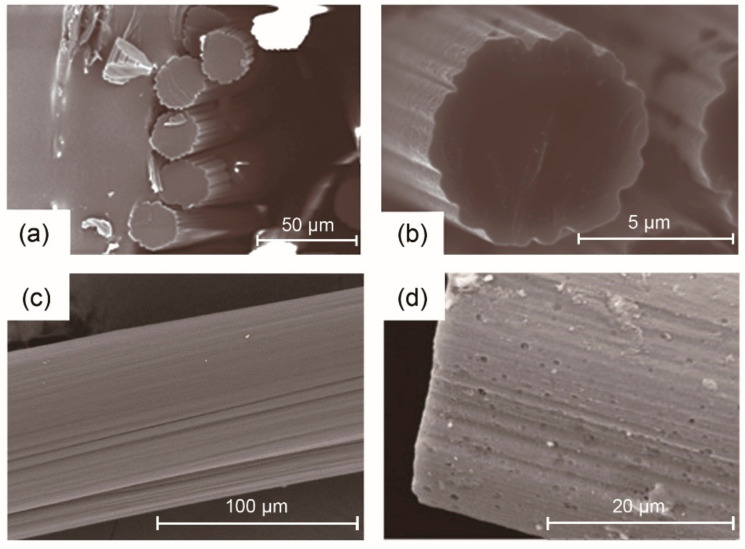
SEM images of (**a**) acetylated softwood Kraft lignin as-spun fibers and (**b**) corresponding carbon fibers by dry-spinning; (**c**) lignin/PVA (70/30) fiber and (**d**) corresponding lignin/PVA porous carbon fiber by wet spinning. (**a**,**b**) Reproduced with permission from [31], copyright 2014 Elsevier. (**c**,**d**) Reproduced with permission from [138], copyright 2019 John Wiley and Sons.

In dry spinning, lignin with low T_g_ is not required and the surface fusion problem occurring in melt-spun lignin-based carbon fibers is avoided since no heat (but solvent) is required for fiber spinning. Otani et al. [27] described dry spinning of lignin-based carbon fiber precursor from alkali solutions. Although the obtained carbon fibers had a TS = 800 MPa, structural defects such as pores could be formed due to the residual alkali in high-temperature treatment. Zhang et al. [31,32] explored the possibility of dry spinning partially acetylated softwood lignin in acetone solution for carbon fiber precursors and discussed the effect of different factors such as spinning temperature and solution concentration on the processability and morphology of lignin-based fibers. The cross-sections of the as-spun fibers and carbon fibers (Figure 8a,b) showed that the fibers were slightly noncircular with surface crenulations originated from the rapid evaporation of acetone. Carbon fibers with smoother crenulations exhibited an optimal TS = 1.04 GPa and TM = 52 GPa. However, this method required a long time for thermo-oxidative stabilization to prevent the fiber fusion problem with a very low heating rate of 0.01 °C/min. Thus, it is not suitable for the large-scale production of low-cost carbon fibers. To increase the stabilization rate of carbon fibers, fiber precursors were further treated with UV irradiation [116]. With slightly decreased mechanical properties of carbon fibers (TS = 900 ± 100 MPa and TM = 34 ± 2 GPa), the stabilization time was significantly reduced from 40 h to 4 h. Moreover, it is found that lignin purity and molecular weight play important roles in the dry spinning process of lignin-based fibers. Fractionated lignin with high purity and high number-averaged molecular weight (M_n_) up to 28,600 Da had good spinnability and good thermal reactivity for rapid stabilization. The dry-spun lignin-based carbon fibers demonstrated excellent mechanical performance of TS = 1.39 GPa and TM = 98 GPa [88].

The enhancement of dry-spun, lignin-based carbon fiber mechanical properties includes the employment of high-purity and high-molecular-weight lignin to diminish structural defects, the chemical modification of lignin to yield a more linear architecture to facilitate molecular orientation during spinning, and the application of tension during heat treatment. In addition to lignin modification, parameters for fiber spinning and heat treatment are vital to achieve lignin-based carbon fibers with small diameter, good spinnability, and ideal mechanical performance. This may require additional efforts in optimizing the spinning process for fibers with satisfactory performance.

Lignin’s relatively low molecular weight and amorphous structure render it difficult to be converted into fibers by wet spinning or gel spinning since these spinning techniques prefer high-molecular-weight raw materials with long, linear structure and adequate extensional viscosity. On the basis of this principle, there have been attempts to develop modified lignin-based wet-spun carbon fiber precursors via copolymerization or grafting. Maradur et al. [119] copolymerized hardwood lignin and acrylonitrile (AN) via a two-step radical polymerization process. The copolymer was successfully wet-spun and converted to carbon fibers with no visible voids in the fiber cross-section. Similarly, butyrated softwood Kraft lignin and organosolv lignin were used to copolymerize with PAN and further wet-spun into precursor fibers [142]. However, neither research mentioned the mechanical properties of the obtained carbon fibers. Alternatively, lignosulfonate–AN copolymer was successfully prepared via a designed two-step process consisting of esterification and free-radical copolymerization [131]. The wet-spun precursor fibers had dense structure with no voids or defects, which yielded carbon fibers with an average TS of ~540 MPa. Similar work was performed to fabricate wet-spun carbon fiber precursors from copolymerized lignosulfonate–AN of different molecular weights [132]. The resulting carbon fiber possessed large diameters (19–35 μm) and an average strength of ~650 MPa.

Chemical modification of lignin by copolymerization greatly enhances the spinnability of the solvent-based solution due to the good dispersion of raw materials in solvent, as well as the higher molecular weight and more linear structures of the copolymers. However, the optimization of processing parameters during spinning, stabilization, and carbonization (i.e., spinning temperature, as-spun draw ratios, fiber draw ratios, heating rate, etc.) of wet-spun modified lignin-based carbon fiber precursors have not been fully investigated. Thus, the advantage of a more linear structure of copolymer has not been not thoroughly exploited to obtain structurally more oriented and finer fibers with better mechanical performance.

##### Solution-Spun Lignin/Polymer Carbon Fibers

More recently, there has been a growing interest in preparing carbon fibers from lignin/PAN blends by solution spinning. Lignin/PAN blends combine unique properties from both polymers such as good spinnability of PAN, high char yield, and bio-renewability of lignin. Specifically, lignin’s polydispersity often causes poor spinnability [29]. This is overcome by blending with PAN polymer, which has a linear long polymer chain, higher molecular weight, and narrower PDI. Continuous carbon fiber precursors from lignin/PAN blends by conventional wet spinning [143] were converted into carbon fibers with a TS of ~2.24 GPa and TM of ~217 GPa [133]. However, when lignin contents were above 20%, voids occurred and the mechanical performance deteriorated [144]. Hollow wet-spun lignin/PAN fibers are formed due to the incorporation of lignin altering the diffusion rate [145,146] and causing phase separation between lignin and PAN [147]. To yield void-free lignin/PAN-based wet-spun fibers, it is important to understand how the processing conditions (i.e., rheological behavior, solubility, etc.) [134,148,149] influence the precursor fiber properties (i.e., morphology, structure, and chemistry of the fibers) since precursor quality (spinning process, structural or morphological defects, etc.) greatly determines carbon fiber quality [37]. For instance, aqueous coagulation baths containing lower than 50% dimethylformamide (DMF) concentration could be suitable for wet spinning of low-molecular-weight lignin/PAN fibers. Lignin concentration has more impact on the orientation of wet-spun lignin/PAN fibers in comparison with spinning rate and coagulation concentration [117].

In general, wet-spun lignin/PAN-based carbon fibers have a unique disordered carbon structure due to the addition of lignin. The mechanical performance of resulting carbon fibers is affected by lignin content, orientation degree, and carbonization temperature. A wheat straw lignin was blended with a commercial textile-grade PAN polymer, and then wet-spun into a water coagulation bath for the production of carbon fibers. However, the derived carbon fiber’s mechanical performance was not satisfactory with TS being only 300–500 MPa and TM being below 100 GPa [144]. The voids in the lignin/PAN blend fibers, which often occurred during wet spinning, were eliminated by adding lignin to the low-temperature (−50 °C) coagulation bath of 65/35 (*w/w*) dimethyl sulfoxide/deionized water (DMSO/DI water) to counterbalance the out-diffusion of lignin. Carbon fibers from 50/50 (*w/w*) lignin/PAN fibers had a TS = 1.2 GPa and TM = 130 GPa [135]. Similarly, wet-spun lignin/PAN-based fibers carbonized at 1200 °C presented a dense structure without any visible macrovoids [150]. The derived carbon fibers had TS = 2.1 GPa and TM = 224 GPa. Unfortunately, the mechanical properties of carbon fibers are primarily attributed to PAN, with little contribution from lignin, and the optimization of draw ratio of wet-spun fibers has been rarely reported.

**Figure 9:**
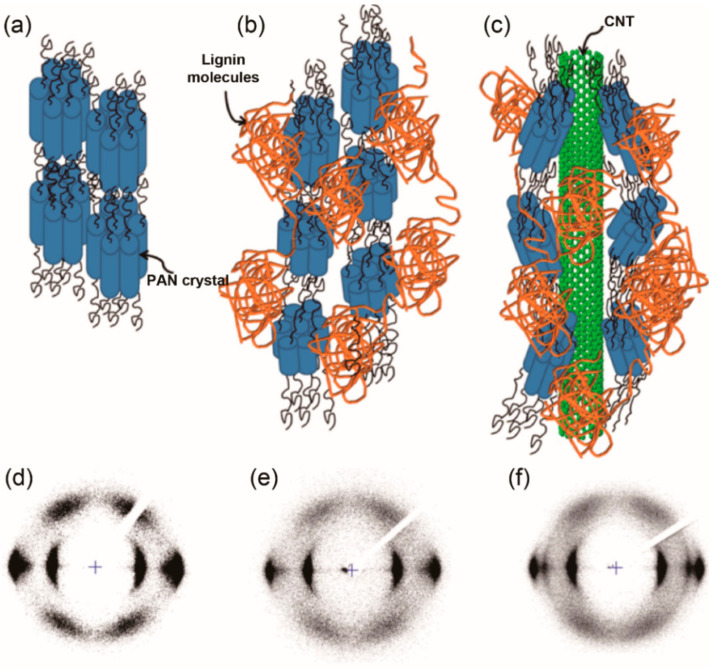
Schematics of gel-spun fibers of (**a**) PAN, (**b**) lignin/PAN, and (**c**) lignin/CNT/PAN fibers. Wide-angle X-ray diffraction (WAXD) patterns of precursor fibers (draw ratio of 13) of (**d**) PAN, (**e**) lignin/PAN, and (**f**) lignin/CNT/PAN fibers. (**a**–**f**) Reproduced with permission from [123], copyright 2015 American Chemical Society.

In addition, gel spinning has also been used to fabricate lignin/PAN blended fibers [123]. The as-spun lignin/PAN (30/70 *w/w*) fibers were obtained from a low-temperature (−50 °C) methanol coagulation bath, stored in a methanol bath at −50 °C for over 12 h, and then further drawn with a high ratio of 13 before being converted into carbon fibers. Compared with pure PAN-based carbon fibers, the lignin/PAN-based carbon fibers exhibited noticeable improvements in TS (1.72 GPa) and TM (230 GPa) with no observable voids. The incorporation of lignin has a positive influence on PAN polymer chain packing, fiber stabilization, and carbonization behavior, as well as the fiber mechanical properties. Composite fibers from lignin, polyacrylonitrile (PAN), and CNTs were successfully fabricated by gel spinning and carbonized into carbon fibers [123]. The PAN/lignin/CNT-based carbon fibers exhibited significant mechanical properties (TS = 1.4 GPa, TM = 200 GPa). In comparison with PAN fiber (Figure 9a,d), the decreased tensile strength was possibly due to the lower PAN orientation (Figure 9b,c) and that PAN crystalline aggregates were disturbed by lignin and CNTs, which resulted in lower crystallinity of lignin/PAN and lignin/CNT/PAN fibers (Figure 9e,f). Furthermore, PAN-sheath and lignin/PAN-core carbon fibers were successfully fabricated using a bicomponent gel spinning technique with maximum draw ratio of 20. The carbon fibers from bicomponent precursors had maximum values of TS = 2.1 GPa and TM = 274 GPa [126], which were comparable to those of PAN-based carbon fibers. Similar to wet-spun fibers, the mechanical properties of gel-spun fibers are related to several processing parameters, including solvent type for dissolution, temperature of the coagulation bath [151], drawing temperature, and total drawing ratio [152]. Thus, it is necessary to optimize the processing condition in order to achieve fibers with the best microstructure and properties.

Cellulose is another common polymer blended with lignin to fabricate 100% renewable high-yield carbon fibers by solution spinning techniques. Cellulose-based precursor fiber alone often has good molecular orientation but low carbon yield (10–30%) due to different degradation reactions which yield pyrolysis gas such as CO_2_, CO, and other low-molar-mass carbon compounds in the carbonization process [139]. A number of stabilization and carbonization protocols [130,136,140,153,154] have confirmed the feasibility of carbon fiber production from wet-spun lignin/cellulose precursor fibers. The optimal lignin/cellulose-based carbon fibers had TS = 1.07 GPa, TM = 76 GPa, diameters less than 10 μm, and a shorter stabilization time (<2 h) [153]. The effects of processing parameters (i.e., draw ratio, stabilization/carbonization temperature, lignin content, etc.) on the structure and mechanical properties of lignin/cellulose precursor fibers and carbon fibers have been investigated systematically [139,155,156,157], from which it is concluded that the cellulose constituent dominates precursor fiber structure and mechanical performance. Increasing lignin content decreases fiber strength due to the disturbance of oriented cellulose crystallites [141,158,159] and increases carbon yield [160] due to lignin’s carbon-rich structure. However, the draw ratio of solution-spun lignin/cellulose precursor fibers seemed to have no significant influence on the carbon fiber’s physical properties [155], although the detailed reasons from the perspective of fiber structure were not revealed. Thus, it is of great importance to achieve precursor fibers with good properties and to optimize the carbonization and stabilization conditions to yield lignin/cellulose-based carbon fibers of good quality.

In addition to the abovementioned investigations, a preferred orientation was introduced by the incorporation of graphene oxide (GO) into lignin [137] to achieve wet-spun precursor fibers and carbon fibers with higher graphitic structure [161]. However, it was found that GO had little contribution to the reinforcement of either precursors or carbon fibers processed at high temperature due to porosity [161]. Similarly, carbon fibers wet-spun from lignin/PVA blends (Figure 8c) showed a porous structure (Figure 8d), which corresponded to poor mechanical performance [138].

In summary, solution-spun lignin/polymer carbon precursor fibers have good spinnability due to the presence of long-chain polymers in the spinning dope and high carbon yield from lignin. However, more efforts should be made in altering lignin structure in order to enhance lignin orientation along precursor fibers to achieve better mechanical performance. Moreover, structure–property relationships should be emphasized systemically in the investigation of fiber solution spinning, stabilization, and carbonization processes to yield fine carbon fibers with few structural defects.

#### 3.1.3. Electrospun Lignin-Based Carbon Nanofibers

Electrospinning is a well-developed and low-cost approach to fabricate continuous fibers at micrometer to nanometer scale [162,163,164,165,166,167,168,169]. With the fast evaporation of solvents, the voltage-driven polymer solution jet is formed, solidified, and collected by the grounded collector (Figure 3e). Lignin-based carbon nanofibers (CNFs) with diameters of 200 nm via electrospinning were first reported in 2007 [170]. It is generally difficult to electrospin a pure lignin solution because lignin cannot form enough chain entanglements within the solution, which often results in electrospray. The spinnability and fiber quality of lignin-based fibers are affected by factors such as solution properties (such as concentration, molecular weight, viscosity), processing parameters (including the solvent type, feeding rate, spinning distance, voltage, and temperature) [171,172,173,174], and apparatus design (i.e., coaxial spinning, rotating collector, etc.) [175,176].

It is highly attractive to produce electrospun fibers with good mechanical performance by tailoring the fiber structure at multiscale (from nanoscale to microscale) [177]. The main limitations of electrospun lignin-based fibers are low yield, low strength, and fiber fusion during the spinning process. Moreover, it is quite difficult to measure the mechanical properties of electrospun single nanofiber since extremely tiny loads for fiber deformation, as well as careful handling of the nanofibers, are required. Alternatively, limited data has been reported on the mechanical properties of electrospun lignin-based nanofiber films or mats, which are obtained either by physical blending of lignin with other polymers or by chemical modification [170,178,179] of lignin for different applications, especially for nanoscale carbon fibers in energy fields [180].

##### Structurally Modified Electrospun Lignin-Based Carbon Nanofibers

Different modifications have been applied to lignin for better electrospinnability. Electrospun submicron platinum (Pt)-doped lignin-based CNFs were prepared [164,181]. Phosphorus-functionalized lignin-based electrospun fibers were converted into CNFs with diameter of 400–1000 nm and mechanical performance (TS = 303 MPa and TM = 4 GPa) [182] comparable to that of melt-spun lignin-based fibers. Fractionated lignin was dissolved in different solvent systems (i.e., dimethylacetamide (DMAc), DMF, etc.) to investigate the optimum condition for mass production of needless electrospun lignin-based CNFs potentially for the electronics industry [183]. You et al. [184] produced organosolv lignin CNFs by reacting with hexamine to crosslink fibers in the stabilization process. The produced CNFs had structural integrity and high specific surface area, as well as a significant percentage of mesopores. Schlee et al. [185] obtained CO_2_-activated Kraft lignin-based CNFs without any other binder polymers or additives. The specific surface area, pores, and the graphitic domains of CNFs significantly increased, which contributed to the improved electrochemical performance. Due to the porous nature of lignin-based CNFs, the main application was focused on sustainable and innovative electrode materials for flexible high-performance supercapacitors [186]. Thus, the mechanical performance of these electrospun modified lignin-based CNFs has rarely been reported.

##### Electrospun Lignin/Polymer Carbon Nanofibers

The polymers blended with lignin to form nanosized carbon fibers by electrospinning are similar to those used in melt spinning or solution spinning. However, the mechanical performance of electrospun lignin/polymer blends-based carbon micro- and nanofibers is generally much inferior to that of conventional fibers [44].

PEO was blended with different types of lignin (ethanol/organosolv, formic acid/acetic acid lignin and Kraft lignin) at a mass ratio of 95/5 to investigate the carbonization behavior of electrospun fibers [187]. It was found that fibers with low-molecular-weight ethanol/organosolv lignin could not be stabilized as they melted, while the other two types of lignin resulted in defective carbon fibers with poor mechanical performance due to the abundant side chains in lignin structure. Kraft lignin yielded carbon fibers with a higher degree of graphitization and better mechanical properties (Table 3) due to better molecular orientation and fewer side chains. Moreover, fractionated lignin with higher molecular weight, narrower PDI, and a more linear structure effectively enhanced the mechanical properties of electrospun carbon fibers (TM = 40 MPa and TS = 6.1 GPa) [188]. Incorporation of 5 wt.% nanocrystalline cellulose (NCC) into lignin/PEO yielded bead-free electrospun CNFs with TS of ~40 MPa and TM of ~6.7 GPa [189], although the presence of NCC had no significant influence on fiber performance.

PAN is the most commonly used polymer for the fabrication of lignin/polymer blend-based electrospun CNFs [196] due to its good spinnability, high molecular weight, and potential to yield high-strength carbon fibers. Butyrated lignin was used as a plasticizer to promote the thermal mobility of electrospun lignin/PAN carbon fibers in a high-temperature treatment process [169]. The butyrated lignin has lower T_g_ since the hydroxyl groups are converted into butyl esters (Figure 4c). The inter-fiber bonding resulted in modified lignin/PAN-based carbon fibers with enhanced mechanical performance (TS = 83 MPa, TM = 6.1 GPa) compared to unmodified ones (Table 3, TS = 22 MPa, TM = 2.1 GPa). Lignins from different extraction processes had various structures, which affected the mechanical performance of CNFs [195]. CNFs from soda lignin/PAN had fewer defects and higher mechanical properties (TM = 142 MPa, TS = 10 GPa) than Kraft lignin/PAN-based CNFs (TS = 99 MPa, TM = 8.6 GPa) due to the more linear structure of soda lignin. Furthermore, fractionated lignin/PAN CNFs had a modulus (21.8 GPa) comparable to commercially available carbon fibers (10–25 GPa) [30].

Iodine treatment was employed before the stabilization of lignin/PAN nanofibers to form charge transfer complexes with aromatic rings [194]. This allowed a higher heating rate (2 °C/min) to be used and facilitated the formation of CNFs with fewer defects, higher graphitization degree, and good mechanical performance (TS = 89 MPa, TS = 5.3 GPa). Dai et al. [197] added graphene into lignin/PAN-based CNFs to enhance the Young’s modulus (2.82 GPa), increase the degree of graphitization, and lower the hydrophobicity. Modified lignin was also blended with PAN to improve its properties. Lignin-grafted-PAN copolymer alone yielded CNFs with a TS of ~89.4 MPa [163], and it was also used as a compatibilizer between lignin and PAN to increase the specific tensile strength of the CNFs to ~160 GPa·mm^3^/g and the Young’s modulus to ~14,000 GPa·mm^3^/g [198]. The spinnability of catalytic depolymerized lignin bio-oil/PAN solution by electrospinning has also been investigated [199]. The small-molecule lignin bio-oil with high reactivity and low heterogeneity facilitated molecule orientation. The best electrospun CNFs were obtained by blending a solution ratio of 80 wt.% lignin and 20 wt.% PAN, with TS = 32.76 MPa and TM = 4.78 GPa.

PVA has also been also blended with lignin to prepare electrospun CNFs. However, the oxygen-rich structure of PVA often results in low carbon yield (<10 wt.%) and microporosity [200] in the high-temperature treatment process. A twisted lignin/PVA CNFs-based yarn was fabricated after electrospinning with rotating motor assembly to introduce a gradual increase in mechanical properties due to the elimination of fiber voids and decreased porosity [201]. The highly twisted fibers exhibited TS = 526.3 MPa, which was much higher than that of the untwisted ones (TS = 48.5 MPa). Although a lot of work has been reported on the electrospinning of lignin/PVA CNFs, most studies focused on other performance, such as electrochemical performance instead of mechanical properties.

Apart from PEO, PAN, and PVA, other polymers such as cellulose acetate [202], cellulose [203], polyvinylpyrrolidone (PVP) [204], poly(methyl methacrylate) (PMMA) [205], and PET [162] have been blended with lignin to yield electrospun CNFs for different applications. However, the mechanical performance was hardly reported.

#### 3.1.4. Centrifugal-Spun Lignin-Based Carbon Fibers

Centrifugal spinning is a simple and controllable technique for the fabrication of nanofibers by subjecting the spinning solution to a centrifugal force (Figure 3f), which is different from the electric force of electrospinning. This fiber spinning technique is denoted as rotary jet spinning [206] or force-spinning [207], which can possibly yield lignin-based nanofibers. Different factors, such as fluid viscosity, collecting distance, rotation speed, and structure of spinneret, affect the fiber diameter, morphology, and mechanical properties. Although centrifugal spinning can achieve high fiber productivity, the preparation of carbon nanofibers with lignin is still in its infancy. Recently, the spinning parameters of lignin-based nanofibers via centrifugal spinning from lignin/thermoplastic polyurethane polymer blends were optimized to obtain thermally stable nanofibers with diameters below 500 nm [208]. However, more investigations need to be conducted.

### 3.2. Lignin as Fiber Reinforcement

Traditionally, one- (1D) and two-dimensional (2D) fillers are used to reinforce polymeric materials. 1D fillers, like carbon nanotubes (CNTs), are popular due to their inherent high strength (50–150 GPa) and high modulus (1 TPa) [209,210]. Graphene sheets are 2D fillers with superior mechanical properties (ultimate TS of ~130 GPa and TM of ~1 TPa) [211].The possibility of utilizing 3D lignin as a filler for the reinforcement of the polymer in the forms of films [212,213,214], hydrogels [215], fibers [216], etc. for different applications provides a unique understanding of polymer reinforcement. This section summarizes lignin-reinforced polymeric fibers regarding their spinnability and mechanical properties.

Lignin has been investigated as a filler for biobased fibers [81]. Melt extruded lignin/PP and lignin/PE fibers show significantly reduced spinnability and mechanical properties with increasing lignin content. The same phenomenon has been observed in lignin/PAN fibers [117], which are typically obtained by wet spinning [217]. Dong et al. [118] blended lignosulfonate (LS)/PAN to prepare fibers via wet spinning. PAN and LS have good miscibility due to the sulfonyl groups in LS molecules and the adsorption of LS onto the macromolecular chains of PAN. As a result, there was no phase separation in the composite fibers. The LS/PAN (47/53 *w/w*) blend fibers had low mechanical performance with TM = 0.72 GPa and TS = 15.45 MPa. Textile-grade lignin/PAN fibers from dry-jet wet spinning developed homogeneous and smooth surfaces with a draw ratio up to 10, fineness values of ~6 dtex, TS of ~616 MPa, and TM of ~30 GPa [218]. Composite fibers have lower mechanical performance than pure PAN fibers due to lignin disrupting the PAN molecular arrangement and decreasing nitrile interactions between PAN polymer chains. Studies about wet-spun lignin/PAN hollow fibers at different ratios have indicated that porosity increases firstly and then reduces as the draw ratio increases, and that it is important to use as-prepared spinning dopes with good homogeneity to increase fiber spinning stability [219].

In summary, the dramatic decrease in mechanical properties of wet-spun lignin/PAN fibers is caused by the decrease of the crystallinity and orientation. The amorphous structure of lignin may disturb or hinder the growth of crystalline domains as a function of its random distribution among PAN chains. Therefore, PAN crystallites are randomly oriented within the fiber. Moreover, fibers with higher lignin content have lower orientation, which further strengthens the hypothesis that the addition of lignin constrains the formation of a more homogeneously ordered structure. Obvious reductions in mechanical performance observed in fibers with high lignin content can be interpreted as a sign of low axial orientation of polymer chains in fiber structure.

Nevertheless, lignin has the potential to reinforce polymers since it is compatible with polar polymers due to its intrinsic polar structure [220]. Kubo et al. [221] claimed that the strong intermolecular interactions between lignin and PVA resulted in good miscibility between the two materials. The spinnability of composite fibers with short-chain and long-chain PVA by melt extrusion were investigated. Short-chain PVA had better spinnability than long-chain PVA due to the lower viscosity. However, mechanical performance was not reported to support the statement of strong interactions between lignin and PVA. Lu et al. [124] reported 5% lignin/PVA gel-spun fiber with values of maximum TS = 1.1 GPa, TM = 37 GPa, and toughness of ~17 J/g, which were much higher than those of neat PVA fibers. The enhancement of fiber performance was attributed to intermolecular bonding between lignin and PVA, a high index of crystallinity, and mild alignment of lignin upon fiber spinning. Intermolecular hydrogen bonding between PVA and lignin enabled better alignment of lignin’s molecular groups as PVA polymer chains were highly stretched along the fiber axis during drawing. However, fibers with higher lignin content had lower mechanical properties due to the voids, lignin aggregation, and poor chain alignment in the fiber structure. To further improve the mechanical properties and molecular anisotropy, Lu et al. [125] examined the effects of gel aging on fiber structure and properties. The 5% lignin fibers had the highest TS of ~1.4 GPa from 1-day gel aging, as well as maximum Young’s modulus of ~54 GPa and toughness of ~25 J/g after 14-day aging. Water in the aging solvent, as a plasticizing agent, aided the swelling of the gel microstructure and resulted in a higher fiber draw ratio of 5% lignin fibers. Moreover, ternary systems of lignin/PVA-based gel-spun fibers with a second filler such as glucarate [222] or GO [223] were investigated. The intermolecular interaction between the polymer matrix and fillers has great impact on the microstructure and further mechanical performance of the obtained fibers.

Ma et al. [224] utilized Kraft and organosolv lignins as fillers to fabricate cellulose-based composite fibers using a dry-jet wet spinning method. It is claimed that fiber spinnability and strength highly depend on the lignin content and spinning dope concentration. The increase in dope concentration to 20 wt.% (if the cellulose/lignin ratio is 1:1) and low spinning temperature enhanced the spinnability. Among lignin-reinforced cellulose fibers, 10% Kraft lignin/cellulose fibers had the highest TS of ~700 MPa and TM of ~24 GPa. However, higher lignin content leads to lower mechanical properties due to lower crystallinity. It is also implied that organosolv lignin is possibly a better filler than Kraft lignin due to the former having higher chemical homogeneity (sulfur-free) and thermal stability, as well as a lower tendency of fragmentation. Similarly, lignin/cellulose fibers with up to 50% lignin were achieved by dry-jet wet spinning [225]. The investigations of the fibers indicated a core/shell structure with a dense core and a porous shell at lower lignin content. It was concluded that cellulose governed fiber formation and final properties while lignin served mainly as a filler in the core region. Spinnability decreased with increasing lignin content due to the decreasing maximum draw ratio, as did the mechanical properties of fibers. Moreover, it was found that lignin increased the maximal critical draw ratio of a cellulose solution with 50% lignin and improved the spinning stability [226]. However, other factors such as spinneret geometry and choice of coagulant remain to be investigated.

It should be noted that lignin tends to leach into the coagulation bath of solution-spun lignin-containing fibers [124,136,225,227], which may affect the solid content and performance of the final fibers. Apart from selecting the appropriate coagulation solvent [124], lignin leaching was lessened by the incorporation of modified lignin into wet-spun cellulose-based fibers, which favored the production of biobased fibers with more lignin retained in fiber structure [228]. The modified lignin had fewer hydroxyl groups due to the enzymatic radical oxidation reactions of lignin with white-rot fungus *Obba rivulosa* increasing the content of aromatic rings connected by carbon/carbon (C–C) bonds. Moreover, spinnability of lignin/cellulose wet-spun fibers decreased as the lignin content went up. By optimizing the draw ratio of wet-spun fibers, both carbohydrate fraction and lignin moieties were highly orientated along the fiber axis, although a slight decrease in TS was observed at higher lignin content.

As for electrospun lignin-based polymeric fibers, the main focus has been the optimization of lignin/polymer nanofibers for bead-free fibers by forming intermolecular interactions between lignin and polymers, such as PEO [179], PVA [229,230,231,232], and cellulose acetate [233]. The properties (viscosity, electroconductivity, and surface tension) of the lignin/PVA dispersion with different loadings of cellulose nanocrystals (CNCs) were correlated with electrospinnability [229,230,231]. The intermolecular interaction of hydrogen bonding among the polymer matrix, lignin, and the dispersed CNCs plays an important role in improving the spinnability and thermomechanical properties of composite electrospun fibers. Moreover, different surfactants (anionic, cationic, and nonionic surfactants) were introduced to yield bead-free lignin/PVA nanofibers with reduced fiber size by decreasing the surface tension of spinning dopes and increasing polymer entanglements [232]. To improve the miscibility of lignin and cellulose acetate, lignin–polyester copolymers were synthesized before electrospinning [233]. It was shown that the incorporation of lignin copolymers into cellulose acetate fibers enhanced the mechanical performance of the material with maximum TS = 8.9 ± 2.4 MPa and TM = 471.0 ± 56.4 MPa due to better alignment and higher crystallinity. These researches highlight lignin’s great potential to be further used or examined as a fiber for load-bearing applications.

## 4. Future Directions

**Figure 10:**
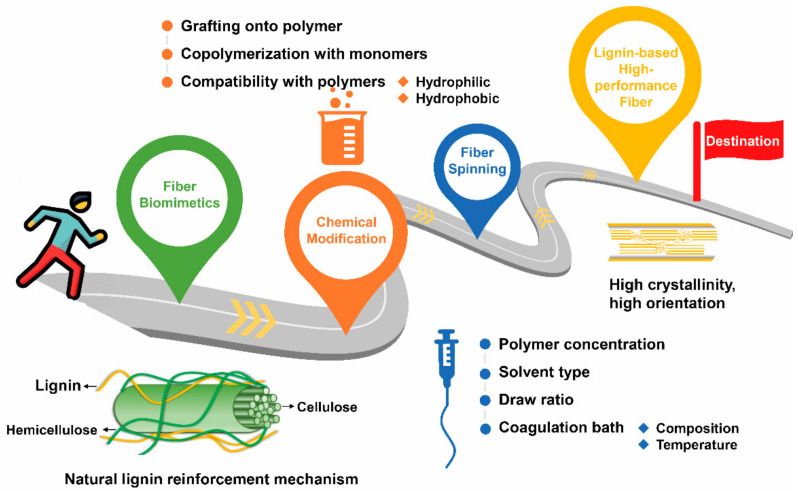
Roadmap of future research directions of lignin-based high-performance fibers.

Even though lignin fibers have been manufactured by various textile spinning techniques over the last 50 years, there are still many difficulties in providing lignin-based fibers with satisfactory physical properties for industrial applications, mainly due to its amorphous structure, brittleness, and immiscible properties. To successfully fabricate lignin-based fibers, it is structurally modified or blended with polymers to improve its spinnability. Lignin-based fibers with greater strength require lignin with high purity, high molecular weight, and a narrow PDI. Even though lignin itself is fairly cheap, the preparations of lignin (i.e., extraction, modification, etc.) for fiber spinning can be quite intricate and expensive. Thus, suitable lignin-containing systems that, when processed into fibers satisfy both property requirements and cost objectives, have rarely been demonstrated. Future advances toward lignin-based high-performance fibers (Figure 10) are listed below.

1. Extraction or pretreatment resulting in lignin with different structural chemistries suitable for various fabrication techniques to manufacture lignin-based high-performance fibers should be more systematically studied to yield lignin with higher molecular weight and narrower PDI. Although efforts have been made to fabricate lignin/cellulose fibers [224,225,226] that resemble fibrils in plant cell walls, there is still a gap in the fundamental knowledge of the physics and chemistry of natural lignin and how it reinforces or provides strength in wood. The knowledge should be gained to potentially develop fiber biomimetics with good mechanical properties.
2. The reported lignin grafting with CNTs [79] or PAN [163], RAFT polymerization [99], and copolymerization with AN [119,234] showed promising results. The future trend of lignin chemical modification should focus on increasing molecular weight and polymer linearity by grafting lignin onto different polymers or via polymerization or copolymerization of lignin with other monomers. For lignin/polymer blends, lignin modification should not be limited to the reduction of lignin’s hydroxyl groups to be compatible with hydrophobic polymers such as PET and PP [234]. More studies of lignin modification should also be conducted to enhance its compatibility with polar polymers.
3. Lignin fiber spinning research is still in its infancy and requires more efforts in material and spinning technique development. Solution spinning of lignin-based fibers should be more thoroughly investigated as an alternative to traditional melt spinning [41]. Solution-spun fibers have higher molecular orientation along the fiber axis, which is vital to achieve high-strength and high-modulus lignin-based fibers. In particular, the optimization of spinning parameters (e.g., draw ratio, solvents, coagulation bath composition, and temperature) that aid the orientation of molecules should be conducted to achieve fibers with small diameter, high crystallinity, high orientation, and high mechanical properties.

With a better understanding of the role of natural lignin as a reinforcing filler, along with the progress of modification chemistry and the development of fiber spinning technology, it is becoming increasingly likely that lignin will be a promising renewable resource for the manufacture of low-cost high-performance products for applications such as carbon fibers or structure reinforcement.

## Figures and Tables

**Table 1:** Literature summary of melt-spun lignin-based carbon fibers.

Modification/ Fractionation	Lignin/Polymer (Mass Ratio) and Reference	Processing Temperature (°C)	Thermal Treatment Stabilization/Carbonization	Diameter (μm)	Mechanical Properties	
					TS (MPa)	TM (GPa)
Unmodified lignin	Hardwood Kraft lignin [82]	195–228	250 °C, 12~180 °C/h, 1 h, air 1000 °C, 180 °C/h, nitrogen	46 ± 8	422 ± 80	40 ± 11
	Hardwood Kraft lignin/PEO (97/3) [82]	189–228	250 °C, 12~180 °C/h, 1 h, air 1000 °C, 180 °C/h, nitrogen	33 ± 2	458 ± 97	59 ± 8
	Pyrolytic lignin [91]	105/125	250 °C, 0.5 °C/min, 1 h, air 1000 °C, 3 °C/min, 1 h, nitrogen	49 ± 2	370 ± 38	36 ± 1
	Organosolv lignin [92]	210	250 °C, 0.5 °C/min, 1 h, air 1000 °C, 3 °C/min, 1 h, nitrogen	14 ± 1	355 ± 53	39.1 ± 13.3
	Hardwood Kraft lignin/PET (75/25) [33]	130–240	250 °C, 0.2–3 °C/min, 1 h, air 1000 °C, 3 °C/min, nitrogen	34 ± 5	703	94
	Hardwood Kraft lignin/PP (87.5/12.5) [93]	130–240	250 °C, 0.2–3 °C/min, 1 h, air 1000 °C, 3 °C/min, nitrogen	44 ± 5	437	54
	Lignin/PLA (80/20) [80]	220–240	280 °C, 0.25 °C/min, 1 h, air 1000 °C, nitrogen	30–60	159.2	11.6
	Organosolv yellow polar lignin [70]	N/A	250 °C, 0.05 °C/min, 30 min, air Stepwise: 600 °C, 3 °C/min, 5 min; 1000 °C, 5 °C/min, 15 min, itrogen	17.1 ± 1.59	544 ± 96	36.5 ± 2.81
	Alcell organosolv hardwood lignin/TPU (50/50) [94]	155/180/190/180	250 °C, 0.1 °C/min, 1 h, air 1000 °C, 10 °C/min, 0.5 h, nitrogen	31 ± 2	1100 ± 100	80 ± 10
	Organosolv lignin from yellow poplar/organosolv lignin from switchgrass (85/15) [95]	180	250 °C, 0.05, 0.1, 0.2 and 0.5 °C/min, 30 min, air Stepwise: 600 °C, 3 °C/min, 5 min; 1000 °C, 5 °C/min, 15 min, nitrogen	15.7 ± 1.1	747 ± 208	41.8 ± 3.9
	Softwood Kraft lignin/hardwood Kraft lignin (90/10) [96]	140–250	250 °C, 0.2 °C/min, 1 h, air Stepwise: 600 °C, 1 °C/min; 1000 °C, 3 °C/min, nitrogen	21.4	N/A	N/A
Fractionation with methanol; acetylation	Modifed organosolv lignin [97]	130	250 °C, 0.1 °C/min, 1 h, air 1000 °C, 3 °C/min, 1 h, argon	39.1 ± 5.4	454 ± 98	62 ± 14
Repolymerization	Repolymerized pyrolytic lignin [98]	115–120	280 °C, 0.3 °C/min, 1 h, air 1000 °C, 3 °C/min, 1 h, argon	29–50	855 ± 159	85 ± 37
Hydrogenation	Modidified steam-exploded lignin [78]	155–180	210 °C, 0.5–2 °C/min, air 1000 °C, 5 °C/min, 20 min, nitrogen	7.6 ± 2.7	660 ± 230	40.7 ± 6.3
Phenolyzation	Modidified steam-exploded lignin [85]	155–180	210 °C, 0.5–2 °C/min, air 1000 °C, 5 °C/min, 20 min, nitrogen	N/A	455	N/A
Stepwise: acrylation, acetylation, and RAFT polymerization	Modified red oak lignin bio-oil [99]	25–280	250 °C, 7 °C/min, air 1000 °C, 3 °C/min, 1 h, argon	5.1	1700	182
Fractionation with acetic acid (AcOH)	Fractionated softwood acetic acid lignin [100]	N/A	250 °C, 0.5 °C/min, 1 h, air 1000 °C, 3 °C/min, nitrogen	84 ± 15	26.4 ± 3.1	3.59 ± 0.43
Butyration	Butyrated lignin/PLA (75/25) [83,101]	180	250 °C, 0.25 °C/min, 5 h, oxygen 1000 °C, 3 °C/min, nitrogen	122 ± 29	N/A	1.94 ± 0.11
Fractionation with isopropyl alcohol	Fractionated organosolv hardwood lignin/PET (75/25) [102]	170/230/252	250 °C, 0.1 °C/min, 1 h, air 1000 °C, 10 °C/min, 30 min, nitrogen	120 ± 27	N/A	N/A
Grafting with PEG	PEG grafted with softwood lignin [103]	145–172	250 °C, 0.1–0.5 °C/min, 1 h, air 1000 °C, 3 °C/min, 1 h, nitrogen	10.4 ± 1.3	457 ± 188	26.2 ± 13.3
Hydroxypropyl modification	Hydroxypropyl modified kraft hardwood lignin/TPU (50/50) [94]	175/190/200/190	250 °C, 0.1 °C/min,1 h, air 1000 °C, 10 °C/min, 0.5 h, nitrogen	30 ± 1	800 ± 100	66 ± 10
Grafting with CNTs	CNTs grafted with lignin (CNTs-g-L) (0.5/99.5) [79]	225	280 °C, 0.25 °C/min,1 h, air 1000 °C, nitrogen	N/A	289.3	18

PEO: poly(ethylene oxide); PET: poly(ethylene terephthalate); TPU: thermoplastic polyurethane; PLA: polylactic acid; PP: polypropylene; CNTs; carbon nanotubes; PEG: polyethylene glycol; RAFT: reversible addition–fragmentation chain-transfer; TS: tensile strength; TM: tensile modulus; N/A: not available.

**Table 2:** Literature summary of solution-spun lignin-based carbon fibers.

Spinning Technique	Lignin Type/Polymer (Mass Ratio)/Solvent and Reference	Modification/Coagulation Condition	Thermal Treatment Stabilization/Carbonization	Diameter (μm)	Maximum Mechanical Properties	
					TS (GPa)	TM (GPa)
Dry spinning	Lignin/alkaline solution [27]	NA	150 °C, 5 °C/min, 10 h; 700 °C, 10 min	20–30	0.8	N/A
	Modified softwood Kraft lignin/acetone [31]	Acetylation with acetic anhydride	200 °C, 0.2 °C/min; 1000 °C, 4.5 °C/min	~7	1.04	52
	Modified softwood Kraft lignin/acetone [116]	Acetylation with acetic anhydride	UV treatment 250 °C; 1000 °C	N/A	0.9 ± 0.1	34 ± 2
	Softwood Kraft lignin/acetic acid–water (H_2_O) [88]	NA	250 °C, 1 h; 1000 °C	6–7	1.39	98
	Beech organosolv lignin/cellulose (30/70, 50/50)/[DBNH]OAc [130]	NA	Stepwise: 240, 250, 260, 270 °C, line speed of 15.6 m/h, 23 min in each stabilization zone; 450, 600, 800, 1200, 1500 °C, line speed of 15.6 m/h, 5.5 min in each carbonization furnace	4–11	~0.48	~26
Wet spinning	Hardwood lignin/DMSO [119]	Copolymerization with AN Coagulation bath: H_2_O	280 °C, 1 h; 800 °C, 5 °C/min	11	N/A	N/A
	Modified lignosulfonate/DMSO [131]	Esterification with acryloyl chloride, then copolymerization with AN (mass ratio of esterified lignin/AN:30/70) Coagulation bath: DMSO/H_2_O	250 °C, 10 °C/min, 1 h; 1400 °C, 10 °C/min	10–20	1.1	N/A
	Modified lignosulfonate/DMSO [132]	Esterification with acryloyl chloride, then copolymerization with AN (mass ratio of esterified lignin/AN: 10/90, 15/85, 20/80, 25/75,30/70) Coagulation bath: DMSO/H_2_O (60/40 *w/w*), 60 °C	250 °C, 10 °C/min, 1 h; 1400 °C, 10 °C/min, 10 min	19–35	0.65 ± 0.05	N/A
	Wheat straw lignin/PAN (1, 3.53, 5, 8.6, 13.3/20)/DMSO [133]	Coagulation bath: H_2_O	250 °C, 1 °C/min, 0.5 h; 1400 °C, 10 °C/min, 20 min	20–50	0.3–0.5	<100
	Softwood Kraft lignin/PAN (50/50)/DMSO [134]	Coagulation bath: DMSO/H_2_O (65/35 *w/w*), 0.2 wt.% of lignin were added into coagulant bath to control out-diffusion	300 °C, 1 °C/min, 1 h; 1200 °C, 7 °C/min, 1 h	7.0 ± 0.3	1.2	130
	Lignin/PAN (37/20)/DMSO [135]	Coagulation bath: DMSO/H_2_O (60/40 *w/w*), 60 °C	250 °C, 10 °C/min, 10 min; Stepwise: 700 °C, 10 °C/min, 10 min, then 1200 °C, 7 °C/min, 30 min	13.5	2.1	224
	Softwood Kraft lignin/cellulose (70/30)/[EMIm][OAc] [136]	Coagulation bath: H_2_O, 5 ± 2 °C	250 °C, 0.2 °C/min, 1 h; Stepwise: 600 °C, 1 °C/min, then 1000 °C, 3 °C/min	< 10	1.07	76
	Kraft lignin/PVA/GO (66/29/5)/DMSO [137]	Coagulation bath: isopropanol	300 °C, 2 °C/min, 1 h; Stepwise: 500 °C, 2 °C/min, then 1000 °C, 5 °C/min	N/A	0.763	52
	Kraft lignin/PVA (70:30)/DMSO [138]	Coagulation bath: 2-propanol	250 °C; 1000 °C, 5 °C/min	37	0.351 ± 0.108	44.5 ± 9.6
	Softwood Kraft lignin/cellulose (70/30)/[EMIm] [OAc] [139]	Coagulation bath: H_2_O, 5 ± 2 °C	Stepwise: 250 °C, 5 °C/min, 60 min, 600 °C, 1 °C/min; 800, 1000, 1200, 1400, 1600 °C, 3 °C/min	N/A	1.1 (Carbonization at 1000 °C)	77 (Carbonization at 1600 °C)
	Softwood Kraft lignin/cellulose (70/30)/[EMIm] [OAc] [140]	Coagulation bath: H_2_O, 15 °C	Stepwise: 200 °C, 0.2 °C/min, 250 °C, 1 °C/min; Stepwise: 600 °C, 1 °C/min, 1000 °C, 3 °C/min	14–15	0.88	67
	Softwood kraft lignin/cellulose (70/30)/[EMIm] [OAc] [136]	Coagulation bath: H_2_O, 5 ± 2 °C	Stepwise: 200 °C, 0.2 °C/min, 250 °C, 1, 5 °C/min, 1, 5, 10 h; Stepwise: 600 °C, 1 °C/min, 1000 °C, 3 °C/min	6–8	1.07	76
	Lignin/cellulose (25/75)/[EMIm] [OAc] [141]	Coagulation bath: H_2_O, room temperature	Stepwise: 240 °C, 5 °C/min, 30 min; 1000 °C, 10 °C/min, 60 min	92.9 ± 2.7	0.12	5.9
	Lignin/PAN(31/69)/DMF [126]	Coagulation bath: methanol, −50 °C	Stepwise: 265 °C, 3 °C/min, 158, 170, 182, 230, 255 min, 305 °C, 3 °C/min, 10 min; 1000, 1200, 1300 °C, 10 °C/min, 10 min	8–9	2.1 (Carbonized at 1200 °C)	274 (Carbonized at 1300 °C)
Gel spinning	Soda lignin/PAN (30/70)/DMAc [123]	Coagulation bath: methanol, −50 °C	Stepwise: 255 °C, 3 °C/min, 400 min, 315 °C, 3 °C/min; 1100 °C, 5 °C/min,	11.0 ± 1.1	1.72	230
	Soda lignin/PAN/CNT (70/30/3)/DMAc [123]	Coagulation bath: methanol, −50 °C	Stepwise: 255 °C, 3 °C/min, 400 min, 315 °C, 3 °C/min; 1100 °C, 5 °C/min	8.8 ± 0.3	1.4	200

N/A: not available; PAN: polyacrylonitrile: PVA: poly(vinyl alcohol); AN: acrylonitrile; GO: graphene oxide; [EMIm] [OAc]: 1-ethyl-3-methylimidazolium acetate; DMSO: dimethyl sulfoxide; DMAc: dimethylacetamide; [DBNH] OAc: 1,5-diazabicyclo [4.3.0] non-5-enium acetate.

**Table 3:** Performance of electrospun CNFs from unmodified lignin/polymer blends.

Lignin/Polymer (Mass Ratio)/Solvent and Reference	Processing Parameters	Carbonization Temperature (°C)	Diameter (nm)	Tensile Strength (MPa)	Young’s Modulus (GPa)
Lignin/PEO (95/5)/DMF [188]	Voltage: 20 kV Flow rate: 0.8 mL/h	1000	867 ± 212	40 ± 3	6.1 ± 0.3
Lignin/PEO (95/5)/DMF [187]	Voltage: 20 kV Flow rate: 0.5 mL/h Distance: 20 cm	1000	1007 ± 70	15.58 ± 2.10	24.54 ± 3.29
Lignin/PEO (28/0.2)/DMF [190]	Voltage: 15 kV Flow rate: 0.02 mL/min	1000	634 ± 87	32 ± 9	4.8 ± 0.6
Lignin/PEO (95/5–99.9/0.1)/DMF [191]	Flow rate: 0.42 μL/s	1000	465 ± 76	11.64 ± 6.94	2.37 ± 0.78
Lignin/PEO (99/1)/DMF [192]	Voltage: 20 kV Flow rate: 0.01 mL/min Distance: 25 cm	800, 900, 1000	500 ± 150	33.7 ± 6	8.0 ± 1.4
Lignin/PAN (50/50)/DMF [193]	Voltage: 25 kV Flow rate: 0.8 mL/h Distance: 20 cm	1000	190 ± 18	21.21 ± 3	4.64 ± 0.1
Lignin/PAN (50/50)/DMF [169]	Voltage: 15 kV Flow rate: 5μL/min Distance: 20 cm	1000	1920 ± 150	22 ± 1	2.4 ± 0.2
Lignin/PAN (50/50)/DMF [194]	Voltage: 20 kV Flow rate: 1.0 mL/h Distance: 20 cm	1400	N/A	56 ± 2	3.2 ± 0.4
Lignin/PAN (0.25/1)/DMF [195]	N/A	1000	208	142 ± 8	10.0 ± 0.4

N/A: not available; PEO: poly(ethylene oxide); PAN: polyacrylonitrile; DMF: dimethylformamide.

## Data Availability

No new data were created or analyzed in this study. Data sharing is not applicable to this article.
